# Fatigue, bleeding tendency and osteoporosis in an adolescent: a diagnostic conundrum

**DOI:** 10.1093/omcr/omad015

**Published:** 2023-02-27

**Authors:** Salwana Ku Md. Saad, Karniza Khalid, Sinead Glackin

**Affiliations:** Department of Paediatrics, Sligo University Hospital, Sligo, Ireland; Endocrine Unit, Specialised Diagnostic Centre, Institute for Medical Research, National Institutes of Health, Ministry of Health Malaysia, Kuala Lumpur, Malaysia; Department of Paediatrics, Sligo University Hospital, Sligo, Ireland

## Abstract

Scurvy is a rare nutritional deficiency, particularly in developed nations. Sporadic cases are still reported, particularly among alcoholics and malnourished. Herein we present an unusual case of a previously healthy 15-year-old Caucasian girl, who was recently hospitalized for low velocity spine fractures, back pain and stiffness over several months and rash for 2 years. She was later diagnosed with scurvy and osteoporosis. Dietary modifications were instituted together with supplementary vitamin C, supportive treatment with regular dietician review and physiotherapy. Gradual clinical recovery was seen over the course of therapy. Our case highlights the importance of recognizing scurvy even among low-risk populations to ensure prompt and effective clinical management.

## INTRODUCTION

Vitamin C not only plays a vital role in the maintenance of oxidation–reduction (redox) balance, tyrosine metabolism and carnitine synthesis [1], but it also affects the treatment outcome of chronic degenerative diseases, autoimmune diseases and malignancy [2]. The deficiency of vitamin C, termed as scurvy, is the manifestation of clinical syndrome from its deficiency [3].

In scurvy, small blood vessels become fragile and easily rupture causing small bleeding areas around the hair follicles, known as perifollicular haemorrhage—a tell-tale sign of a severe vitamin C deficiency [4]. Apart from that, vitamin C deficiency also causes alteration in the structural collagen, contributes to defective osteoid matrix formation and leads to an increase in bone resorption [5]. In 80% of cases, scurvy manifestations include musculoskeletal symptoms such as arthralgia, myalgia, haemarthrosis or muscular haematomas [5]. Radio-imaging may show osteolysis, loss of joint space, osteopenia or periosteal proliferation, with trabecular and cortical osteoporosis being a common observation [5, 6].

Our article aims to describe a challenging case of a young lady who presented with recent low-energy spinal fractures in the presence of long-standing diffuse perifollicular papules and perpetual fatigue.

## CASE REPORT

A 15-year-old, otherwise healthy, Caucasian girl was referred to us by our orthopaedic colleague for detailed assessment following a T12 wedge fracture caused by a low impact trampoline injury. There were features of osteoporosis and a significantly reduced Z score on a DEXA scan.

**Figure 1:**
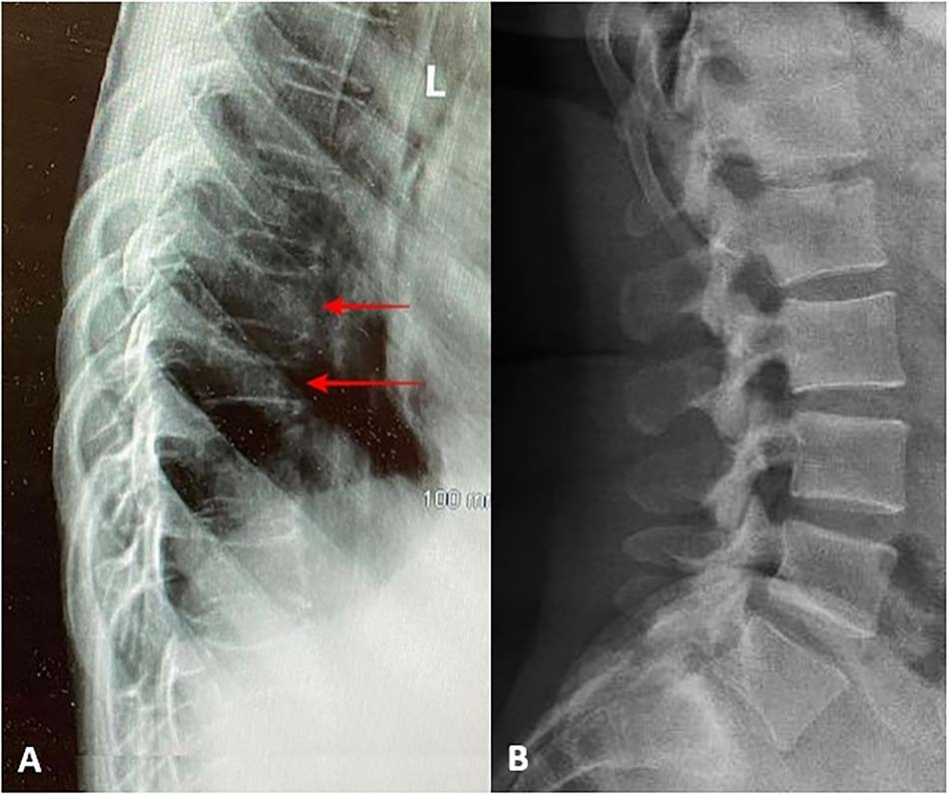
(A) Widespread biconcave and plate depression over thoracic and lumbar vertebrae (red arrows) in comparison with (B) normal thoracolumbar lateral X-ray.

She also complained of progressive fatigue with persistent low back pain and back stiffness for the past 8 months. Thoracolumbar X-ray showed widespread biconcave and plate depression over the thoracic and lumbar vertebrae (Fig. 1), with minor loss of T9 vertebral body height and minor depression of the superior endplate of T8.

**Figure 2:**
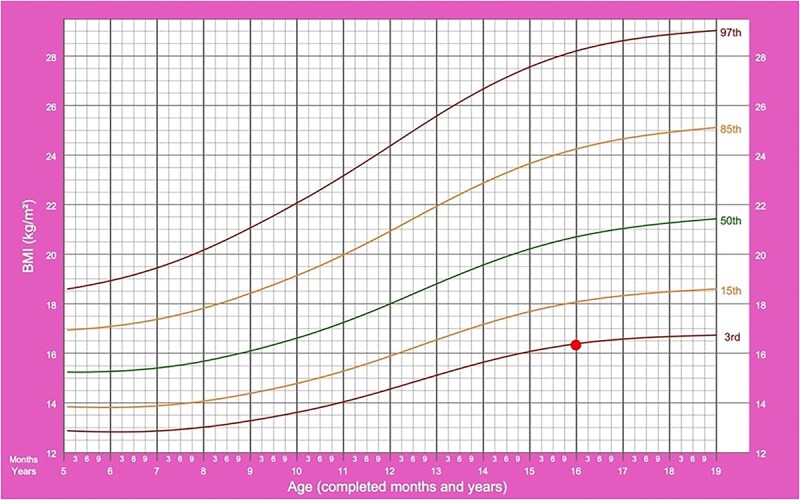
Body mass index percentile chart.

Physical examination noted a slim body habitus with mild kyphosis. Her body mass index was 16.9 kg/m^2^ at third percentile (Fig. 2) with a weight of mere 36.2 kg, well below the fourth percentile for her age and height. In view of the multiple wedge-shaped compression fractures throughout the thoracolumbar region on imaging that were likely to be related to significant insufficiency of the endplate, our orthopaedic team has considered the diagnosis of Scheuermann disease, especially in view of other signs to suggest haematological disorder and no prior history of prolonged steroid therapy. She was then referred to a dietician and was given a review appointment.

**Figure 3:**
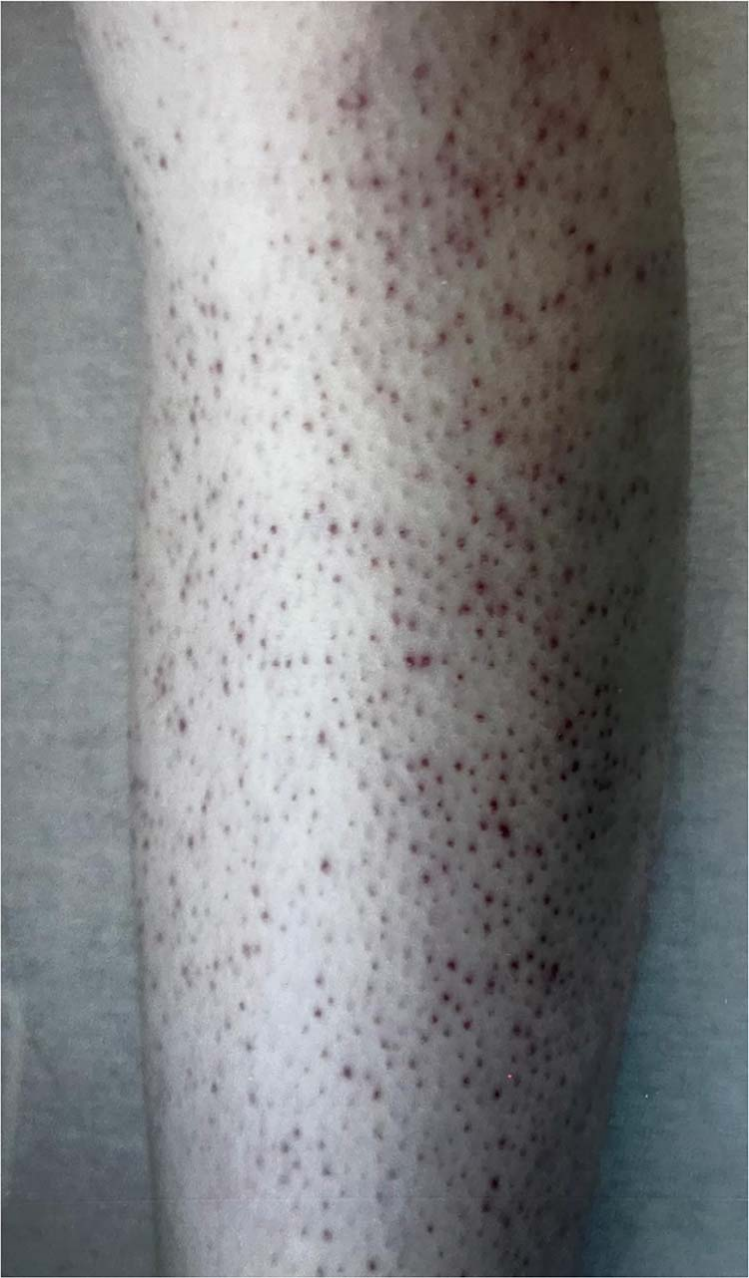
The figure shows diffuse perifollicular haemorrhages and hyperkeratotic papules on left forearm extensors.

However, 5 months later, she was readmitted for lethargy with patches of ecchymoses noted over bilateral knees. Examination also revealed perifollicular purpuric macules over bilateral lower limb and forearms (Fig. 3), claimed to be present since the past 2 years with no abrupt worsening.

There were no joint swelling, joint tenderness, palpable masses or lymphadenopathies on peripheral examination. Other systemic examinations were unremarkable. Her blood pressure was normal for her age and height with a normal resting heart rate.

Our first clinical impression at that point was autoimmune disorders or vasculitis. An extensive work-up was performed (including serum electrolytes, trace minerals and thyroid function) with equivocal results. Her renal, liver functions, coagulation profile, Vitamin B12 and folate level were within normal limits, and Vitamin D level and iron studies were within acceptable range. Her immunological screening with rheumatoid factor and anti-nuclear antibody came back negative. Her erythrocyte sedimentation rate was mildly elevated at 42 mls/h (N:0–20 mls/h) and C-reactive protein was 6 mg/L (*N* < 5 mg/L).

A suspicion for scurvy was only established after a detailed dietary history suggested anorexic behaviour of restrictive type since the age of 10. Vitamin C level was sent and subsequently came back undetectable (*N* = 26–85 umol/L). Her breakfast typically includes two slices of dry toast and plain pasta, with chocolate rice cakes for lunch and tea. She exceptionally lacks red meat, fish, chicken, green vegetables and fruits in her diet. Otherwise, there was no history of prolonged diarrhoea or vomiting, and no history of gastrointestinal diseases in the family. Coeliac disease screening was also negative.

She was prescribed with oral Vitamin C supplement 500 mg daily, ferrous fumarate 1 capsule twice daily (100 mg elemental iron) and calcium carbonate 10 mmol 6 hourly. A referral for psychiatric assessment and psychological services to rule out psychogenic cause of poor dietary habit was made, and she was also seen by a dietician for proper dietary advice and consultation.

## DISCUSSION

We presented a rare case of symptomatic scurvy in a Caucasian adolescent. The case posed as a diagnostic challenge as scurvy is exceedingly rare in a first world country. Our patient was initially referred due to concerns of anorexia nervosa or avoidant/restrictive food intake disorder (ARFID), therefore routine evaluation of vitamin D and calcium level were sent as part of the standard practice. We only sent for vitamin C level when she developed other associated symptoms.

Scurvy is a clinical cutaneous diagnosis presenting with diffuse perifollicular purpura with corkscrew hair in the presence of low Vitamin C level [7]. It is a rare nutritional deficiency and is almost forgotten in developed economies; the disease is commonly associated with malabsorption, malnutrition, alcoholism and old age [8, 9]. The normocytic nonregenerative anaemia associated with scurvy is multifactorial, often coupled with iron, vitamin B9 and B12 deficiencies with evidence of systemic inflammation [10]. Therefore, our case serves as a reminder to the clinicians to be wary of the disease as it is still being sporadically reported. Its clinical appearance can be dramatic, but the disease is readily and completely reversible with restoration of vitamin C levels.

Hence, scurvy should be considered in an appropriate clinical setting, in patients with bleeding tendencies and cutaneous features, after ruling out other haematological conditions. This case highlights that vitamin C deficiency is still relevant in the modern era and raises the need to educate the primary care provider, who are often the first contact, for prompt diagnosis and treatment.

## CONFLICT OF INTEREST STATEMENT

The authors declare no conflict of interest.

## FUNDING

The authors received no financial support for the research authorship and/or publication of this article.

## ETHICAL APPROVAL

The study has been registered with the National Medical Research Register of the Ministry of Health Malaysia (NMRR-21-1392-60 859).

## CONSENT

Written informed consent for publication of the case and photographs used was obtained from the legal guardian.

## GUARANTOR

Ku Md Saad and Glackin will act as the guarantor for the paper.
